# Does Incorporating Gender Differences into Quantifying a Food Frequency Questionnaire Influence the Association of Total Energy Intake with All-Cause and Cause-Specific Mortality?

**DOI:** 10.3390/nu12102914

**Published:** 2020-09-23

**Authors:** Minji Kang, Song-Yi Park, Carol J. Boushey, Lynne R. Wilkens, Loïc Le Marchand, Jean H. Hankin, Hee-Young Paik

**Affiliations:** 1 Center for Gendered Innovations in Science and Technology Research (GISTeR), Korea Federation of Women’s Science & Technology Associations, Seoul 06130, Korea; mjkang@kofwst.org; 2 Cancer Epidemiology Program, University of Hawaii Cancer Center, Honolulu, HI 96813, USA; spark@cc.hawaii.edu (S.-Y.P.); cjboushey@cc.hawaii.edu (C.J.B.); lynne@cc.hawaii.edu (L.R.W.); loic@cc.hawaii.edu (L.L.M.); hjeanh@hawaii.rr.com (J.H.H.); 3 Department of Food and Nutrition, College of Human Ecology, Seoul National University, Seoul 08826, Korea

**Keywords:** portion size, food frequency questionnaire, total energy intake, mortality risk

## Abstract

This study aims to evaluate whether incorporating gender differences in portion sizes as part of quantifying a food frequency questionnaire influences the association of total energy intake with mortality. The analysis included 156,434 participants (70,142 men and 86,292 women) in the Multiethnic Cohort Study, aged 45–75 years at baseline. A total of 49,728 deaths were identified during an average follow-up of 18.1 years. Total energy intake and percentage energy from macronutrients were calculated using original portion sizes (PSs) and gender specific (GS)-PS and were divided into quintiles for men and women. The associations of total energy intake and percentage energy from macronutrients with all-cause, cardiovascular disease (CVD), and cancer mortality were examined using Cox regression with adjustment for potential confounders. Mean ± standard deviation daily total energy intake using original-PS was 2449 ± 1135 kcal for men and 1979 ± 962 kcal for women; using GS-PS was 1996 ± 884 kcal for men and 1595 ± 731 kcal for women. For men, the hazard ratios (HRs) (95% confidence intervals) for all-cause, CVD, and cancer comparing the highest to the lowest quintile of total energy intake were 1.05 (1.00–1.10), 1.07 (0.99–1.16), 1.03 (0.95–1.13) using original-PS and 1.07 (1.02–1.12), 1.11 (1.03–1.20), 1.02 (0.94–1.12) using GS-PS, respectively. For women, the corresponding HRs were 1.03 (0.98–1.09), 0.99 (0.91–1.08), 1.10 (1.00–1.21) using original-PS and 1.06 (1.01–1.12), 1.02 (0.94–1.12), 1.07 (0.97–1.18) using GS-PS. Both versions of percentage energy from total fat were associated with an increased risk of all-cause, CVD, and cancer mortality; on the other hand, both versions of percentage energy from carbohydrate showed inverse associations with all-cause, CVD, and cancer mortality in both men and women. When using original-PS and GS-PS, the estimated total energy intake differed, resulting in marginal differences in the associations of total energy intake with all-cause, CVD, and cancer mortality.

## 1. Introduction

In large-scale epidemiological studies, food frequency questionnaires (FFQs) are primarily used to assess usual dietary intakes. An FFQ can provide estimates of long-term intake by self-reporting and may be less detailed than open-ended methods such as a 24-h dietary recall (24HDR) or a dietary record [1,2]. A quantitative FFQ (QFFQ) consists of a list of relevant food items, choices for frequency of consumption, and portion size (PS) options [3]. An FFQ is usually developed with and validated against 24HDRs or dietary records in target populations [2].

Differences in food consumption between men and women have been recognized by several studies. Men’s food portions were larger than women’s in general, but not for all single foods or food groups compared [4,5,6,7]. There were also gender differences in food choices [8,9,10]. For example, several studies have reported that women eat more fruits, vegetables, cereal products, milk and dairy products than men; whereas men eat more meat products (such as red meat and pork), eggs, alcohol, and various starch foods than women [8,9,10]. Although food portions and preferences vary by gender, our previous review showed that only 10.7% of the 196 FFQs considered gender during the FFQ development process such as food item selection or PS determination [11].

In a validation study of an FFQ administered in the European Prospective Investigation into Cancer and Nutrition (EPIC)-Potsdam Study, two different PSs (predefined PS and sex-specific fitted PS) were applied to FFQ quantification [12]. The estimated values from the FFQ were compared with the values from 24HDRs and the absolute nutrient intakes were closer to the 24HDRs after implementation of sex-specific fitted PS compared with predefined PS, but the effect on the ranking of study participants was marginal [12]. In our previous analysis of the Multiethnic Cohort Study (MEC), gender specific-PS (GS-PS), accounting for gender differences in PS, for the multiple options in the baseline QFFQ was determined [13]. The energy and macronutrient intakes estimated using the GS-PS were closer to the reference method (24HDRs) than the values estimated using the original-PS in both men and women, but there was no difference for percentage energy from macronutrients [13]. However, it is not known whether incorporating gender differences into FFQ quantification affects the associations between dietary factors and health outcomes. Therefore, in the present study, we examined the associations of total energy intake, assessed using the original-PS and GS-PS, with all-cause, cardiovascular disease (CVD), and cancer mortality in the MEC.

## 2. Methods

### 2.1. Study Population

The MEC is a large prospective cohort established to examine the association of lifestyle and genetic factors with the risk of cancer and other chronic disease in Hawaii and Los Angeles [14]. Over 215,000 men and women aged 45–75 years who were primarily of five major race/ethnicities, African American, Native Hawaiian, Japanese American, Latino, and non-Hispanic white, were recruited in 1993–1996 [14]. At cohort entry, participants completed a self-administered comprehensive questionnaire that included a QFFQ and questions on demographics, medical history, reproductive history for women, occupational history, physical activity and consent to participate in the study. The protocols were approved by the Institutional Review Boards at the University of Hawaii (CHS9575) and the University of Southern California (HS-17-00714).

For the present study, participants who did not self-identify as one of the five major racial/ethnic groups (*n* = 13,987) or who reported implausible diets based on total energy intake or its components (*n* = 8241) were excluded. The ranges for implausible energy intake were developed as mean ± 3 robust standard deviation (RSD) where the robust standard deviation (RSD) was computed based on the truncated normal distribution excluding the top and bottom 10% tails of log-transformed energy. Then, all energy values outside of these ranges were excluded. A similar procedure was performed for fat, protein, or carbohydrate intakes to exclude individuals outside the range of mean ± 3.5 RSD. Participants who had a previous history of cancer, heart attack/angina, or stroke at baseline (*n* = 36,982) were further excluded. A total of 156,434 participants (70,142 men and 86,292 women) were included in the analyses.

### 2.2. Dietary Intake Estimated from the QFFQ Using either Original-PS or GS-PS

Dietary data were collected using a QFFQ, which was developed and validated for application in the MEC [14,15]. More than 180 items were listed in the QFFQ. Usual intake over the past 12 months was assessed using eight frequency categories (“never or hardly ever” to “two or more times a day”) for foods and nine categories (“never or hardly ever” to “four or more times a day”) for some beverage items. Quantities of foods were assessed using multiple choices of PS specific to each food item. Participants were able to select a PS for 163 food items on the QFFQ: two PS choices for five food items (e.g., bread spreads), three choices for 151 items, and four choices for seven items (e.g., alcoholic beverages and sodas). Photographs showing different PS (A, the smallest, B, and C or D, the largest) of representative foods were displayed on several pages of the QFFQ. A copy of questionnaire is available on the MEC website [16].

Dietary intake for each study participants was calculated based on the two types of PS value for each food items on the QFFQ: original-PS and GS-PS. The original-PS, a gram amount assigned for each PS category of items on the QFFQ that have been used for the MEC QFFQ, was based on typical PS for each item as reflected in the distribution from three-day measured food records and as described in the questionnaire [14]. Three categories of PSs were provided based on the food consumption distribution that mostly had three common peaks [14,17,18]. For example, mixed dishes were given in specific amounts (e.g., ½ cup, 1 cup, 2 cups) based on the most commonly eaten amounts in grams within the ranges of the three peaks. For food that did not have three common peaks, three portion size options were determined based on percentiles. Integers were provided for countable items, such as eggs. In addition, for beverage items, four portion size options (i.e., ½, 1, 2, 3 cans of soda) were provided. Each portion size was converted to gram weight equivalents.

The GS-PS, a gram amount assigned for each PS category for men and women separately, was determined based on PS distributions for men and women separately from the three 24HDRs in the calibration study of the MEC [13]. For example, among women who selected PS A for the specific food item on the QFFQ, the mean intakes were computed per eating episode reported on the 24HDRs. Then, the median value from the distribution of the average amounts was calculated and used as the gram amount for the GS-PS among women selecting PS category A for this food item on the QFFQ. Overall, about two-thirds of all categories were determined as the gender-specific median values. For items where the distributions were less robust, other methods were used for GS-PS quantification, including values assigned by utilizing ratios to median values (17% for men, 13% for women), by adapting the values of other similar PSs (8% for men, 11% for women), or by using the original-PS (7% for both men and women) [13].

### 2.3. Case Ascertainment

Deaths were identified by linking the cohort to death certificate files in Hawaii and California and the U.S. National Death Index through 31 December 2014. Deaths from CVD were defined by primary cause of death using International Classification of Diseases, Ninth Revision (ICD-9) codes 390-448 or Tenth Revision (ICD-10) codes I00-I78. Deaths from cancer were defined using ICD-9 codes 140-208 or ICD-10 codes C00-C97. All-cause mortality included CVD and cancer death as well as death from other causes, including accidents and suicides. During an average of 18.1 ± 4.9 years of follow-up, we identified 49,728 deaths, including 17,073 from CVD and 14,459 from cancer, among 156,434 eligible participants.

### 2.4. Statistical Analysis

The associations of total energy intake and percentage energy from macronutrients with all-cause, CVD, and cancer mortality were examined using Cox regression with age as the time metric. Hazard ratios (HRs) and 95% confidence intervals (CIs) were calculated for quintiles of total energy intake and percentage energy from macronutrients, and the lowest quintile was used as a reference category. Linear trend was evaluated based on the sex- and racial/ethnic-specific medians within each quintile as a continuous variable. Analyses were adjusted for the following variables at cohort entry: age (<50, 50–54, 55–59, 60–64, 65–69, ≥70 years), race/ethnicity (5 groups), body mass index (<25, 25–29.9, ≥30 kg/m^2^, missing), education (≤12, 13–15, ≥16 years, missing), moderate-to-vigorous physical activity (<0.36, 0.36–0.82, 0.83–1.67, ≥1.68 h/day for men; <0.35, 0.35–0.70, 0.71–1.20, ≥1.21 h/day for women, missing), alcohol intake (0, 0.1–5.1, 5.2–22, ≥23 g/day for men; 0, 0.1–2.4, 2.5–9.9, ≥10 g/day for women), smoking status (never, former, current, missing), and menopausal hormone therapy use (never, former, current, missing) for women only as strata variables. In addition, models for percentage energy from total fat and carbohydrate included total energy intake (log transformed) as a covariate. Separate models were fitted for men and women. All analyses were conducted with SAS statistical software version 9.4 (SAS Institute Inc., Cary, NC, USA) and *p* < 0.05 was defined as significant.

## 3. Results

### 3.1. Participant Characteristics

Mean daily total energy intake using original-PS was 2449 ± 1135 kcal for men and 1979 ± 962 kcal for women; using GS-PS was 1996 ± 884 kcal for men and 1595 ± 731 kcal for women. Although the absolute amount of total energy intake estimated by the original-PS and GS-PS differed, the baseline characteristics of participants according to the quintiles of total energy intake were similar (Table 1). Compared to men and women in the lowest quintile of total energy intake, participants in the highest quintile tended to be younger, Native Hawaiian, Latino, higher in body mass index, less educated, and more physically active. Participants with a higher total energy intake tended to be current smokers among men and less likely to use menopausal hormone therapy among women.

### 3.2. Total Energy Intake and Mortality

Table 2 and Table 3 show HRs (95% CI) for all-cause, CVD, and cancer mortality according to the quintiles of total energy intake estimated by original-PS or GS-PS among men and women. Both versions of higher total energy intakes were associated with an increased risk of all-cause mortality in men (Table 2). The multivariable-adjusted HRs (95% CI) for the highest vs. lowest quintile of total energy intake were 1.05 (1.00–1.10) (*p* for trend = 0.0193) based on the original-PS and 1.07 (1.02–1.12) (*p* for trend = 0.0010) based on the GS-PS. Compared with total energy intake estimated by the original-PS, those estimated by GS-PS showed the same or slightly stronger associations with CVD mortality in men. The HRs (95% CI) of CVD mortality was 1.07 (0.99–1.16) (*p* for trend = 0.0844) in original-PS and 1.11 (1.03–1.20) (*p* for trend = 0.0073) in GS-PS. There were no significant associations between cancer mortality and total energy intake either in the original-PS or GS-PS among men. For women, the HRs (95% CI) for all-cause mortality for the highest vs. lowest quintile of total energy intake were 1.03 (0.98–1.09) (*p* for trend = 0.2704) in the original-PS and 1.06 (1.01–1.12) (*p* for trend = 0.0546) in the GS-PS (Table 3). For CVD or cancer mortality, women did not show an increased risk with higher total energy intake either in the original-PS or GS-PS.

### 3.3. Macronutrient Intakes and Mortality

Medians for each quintiles of percent energy from total fat or carbohydrate were similar between the values estimated by the original-PS and GS-PS (Table 4 and Table 5). For example, medians of the highest quintile for percent energy from fat were 40.5% in the original-PS and 40.9% in the GS-PS among men (Table 4). For men, comparing the highest vs. lowest quintile of percent energy from fat, the HRs (95% CIs) for all-cause, CVD, and cancer were 1.22 (1.16–1.28), 1.27 (1.17–1.38), and 1.16 (1.06–1.26) in the original-PS, and 1.24 (1.18–1.30), 1.30 (1.19–1.41), and 1.17 (1.07–1.27) in GS-PS, respectively. Comparing the highest vs. lowest quintile of percent energy from carbohydrate, the corresponding HRs (95% CI) were 0.81 (0.77–0.85), 0.75 (0.69–0.82), and 0.92 (0.84–1.00) in original-PS, and 0.80 (0.76–0.84), 0.72 (0.66–0.78), and 0.90 (0.82–0.98) in GS-PS, respectively.

For women, comparing the highest vs. lowest quintile of percent energy from fat, the respective HRs (95% CI) for all-cause, CVD, and cancer were 1.11 (1.05–1.18), 1.17 (1.06–1.28), and 1.06 (0.95–1.17) in original-PS, and 1.16 (1.10–1.22), 1.18 (1.08–1.30), and 1.13 (1.02–1.24) in GS-PS (Table 5). The linear trend became stronger and was nominally significant for GS-PS compared to original-PS for cancer mortality in women. The corresponding HRs (95% CI) for percent energy from carbohydrate were 0.85 (0.81–0.90), 0.81 (0.74–0.89), 0.90 (0.81–1.00) in original-PS, and 0.85 (0.81–0.90), 0.81 (0.74–0.88), and 0.91 (0.82–1.00) in GS-PS, respectively.

## 4. Discussion

This study examined whether incorporating gender differences into quantifying a QFFQ influences the associations of total energy intake with all-cause and cause-specific mortality. Mean daily total energy intake using GS-PS was lower than those using the original-PS both in men and women. Marginal differences in the associations of total energy intake with all-cause CVD, and cancer mortality were found using original-PS and GS-PS. When using original-PS and GS-PS, the estimated energy contribution from macronutrients were similar, yielding similar risk estimates.

The energy, derived from the oxidation or breakdown of carbohydrate, protein, fat, and alcohol, is required to sustain the body’s various functions, including respiration, circulation, physical work, and maintenance of core body temperature [19,20]. Energy balance in an individual depends on their dietary energy intake and energy expenditure, and consistent imbalance of energy results in either a loss or gain of body weight [19,20]. In developed countries, increased energy intake in excess of energy need, with resulting obesity, is common and leads to long-term effects of disease incidence such as, type 2 diabetes, cardiovascular disease and a number of other comorbidities [19,21].

Several prospective cohort studies have reported the associations between total energy intake and all-cause or cause-specific mortality [22,23]. Leosdottir et al. reported that individuals approximately meeting Swedish national recommendations for total energy intake had the lowest mortality using data from the Malmö Diet and Cancer Study, which is a population-based prospective cohort study including 28,098 individuals (mean age 58.2 years) [22]. During an average of a 6.6-year period of follow-up, the lowest total mortality was observed for women in the third quartile (mean (range): 2104 (1947–2279) kcal) (relative risk, RR: 0.74 compared to first quartile; 95% confidence interval, CI: 0.57-0.96) and for men in the second (2229 (2043–2414) kcal) and third quartiles (2621 (2415–2859) kcal) (RR: 0.85 compared to first quartile; 95% CI: 0.69–1.04 and RR: 0.85; 95% CI: 0.69–1.04, respectively) [22]. In the NIPPON DATA80 which included 7704 participants aged 30-69 years, Nagai et al., reported a significant association between increased energy intake and all-cause mortality risk in men over 29 years of follow-up (*p* for trend = 0.008) [23]. Cause-specific mortality in the highest quintile was increased for coronary heart disease mortality in men (mean (standard deviation): 3099 (250) kcal) (hazard risk, HR: 2.63; 95% confidence interval, CI: 0.95–7.28, *p* for trend = 0.016) and women (2488 (213) kcal) (HR: 2.91; 95% CI: 1.02–8.29, *p* for trend = 0.0032) [23].

In the present study, using data from the MEC, comparing those with the lowest total energy intake to those with the highest total energy intake, an increased risk of all-cause mortality was 5% in the original-PS and 7% in the GS-PS among men. For women, the corresponding estimation was 6% in the GS-PS and a non-significant 3% in the original-PS. When using the original-PS and the GS-PS, the estimated total energy intake differed; however classification of participants into quintiles by total energy intake with the two versions of PS was not demonstrably different [13]. Thus, differences in the magnitudes of associations with mortality were marginal. However, the hazard ratios estimated by GS-PS were similar or shifted away from the null compared to those using original-PS, resulting in associations becoming significant for CVD mortality in men and all-cause mortality in women.

Dietary guidelines generally recommend a low-fat diet, and diets high in fat have been associated with coronary heart disease and obesity complications, likely due to the saturated fat component [20,24]. Our findings support the current recommendations and observed a higher risk of all-cause mortality, CVD or cancer mortality with higher contributions from total fat to total energy intake; the percentage of energy from fat in this study was higher than the recommended value (median of the highest quintile: original-PS: 40.5% and GS-PS: 40.9% for men, original-PS: 39.5% and GS-PS: 39.3% for women). Most of the participants’ energy contribution from carbohydrate was within the recommended range, and both versions (original-PS or GS-PS) of percent energy from carbohydrate showed inverse associations with all-cause and cause-specific mortality in both men and women.

The present study has several strengths including a prospective design, large number of participants in a population-based cohort, and comprehensive information on a wide range of potential covariates. However, there are several limitations to consider. First, this study examined gender only in terms of the portion size modification and not in the food item selection of the QFFQ. Second, dietary data collected at baseline in a cohort study are subject to non-differential measurement error, generally resulting in attenuated risk estimates [25]. Lastly, although a wide range of covariates were available for adjustment in the models, the possibility of residual or unassessed confounding could not be ruled out.

## 5. Conclusions

In conclusion, the present study suggests that modification of QFFQ portion sizes by gender had lowered estimates of total energy intake, but not in percentage energy from macronutrients in both men and women. In addition, this resulted in marginal differences in the associations of total energy intake and energy contribution from macronutrients with all-cause and cause-specific mortality. For nutritional epidemiological studies using dietary variables adjusted for energy intake (e.g., % energy, nutrient densities), using GS-PS may not be necessary. Further studies are needed to explore the benefits of using GS-PS, especially in studies using absolute intake.

## Figures and Tables

**Table 1:** Baseline characteristics of participants (*n* = 156,434) in the Multiethnic Cohort Study by quintiles of total energy intake estimated from a food frequency questionnaire either using an original-portion size or a gender specific-portion size.

Characteristics at Cohort Entry		Original-Portion Size					Gender Specific-Portion Size				
		Quintile 1	Quintile 2	Quintile 3	Quintile 4	Quintile 5	Quintile 1	Quintile 2	Quintile 3	Quintile 4	Quintile 5
**Men (*n* = 70,142)**	***n***	**(*n* = 13,403)**	**(*n* = 13,842)**	**(*n* = 14,127)**	**(*n* = 14,282)**	**(*n* = 14,488)**	**(*n* = 13,699)**	**(*n* = 13,901)**	**(*n* = 14,056)**	**(*n* = 14,165)**	**(*n* = 14,321)**
Median total energy intake, kcal	70,142	1243.6	1749.6	2191.6	2748.3	3877.3	1066.2	1470.5	1812.3	2236.2	3098.1
Race/ethnicity, %											
African American	9010	20.2	12.4	10.6	9.7	11.7	20.8	12.1	10.5	9.8	11.3
Native Hawaiian	4990	5.1	5.4	6.5	7.3	11.1	5.6	5.9	6.6	7.5	9.8
Japanese American	21,241	27.0	34.8	35.3	32.4	22.1	27.6	35.8	35.7	32.1	20.4
Latino	17,586	25.3	20.6	20.6	23.7	34.8	22.5	19.4	19.9	23.9	39.3
Non-Hispanic white	17,315	22.5	26.8	27.0	26.9	20.3	23.5	26.8	27.3	26.7	19.1
Age groups, %											
45–54 years	24,345	27.2	32.1	34.7	37.6	41.3	31.7	34.4	34.3	36.0	37.0
55–64 years	23,617	34.0	33.5	32.4	33.5	34.8	34.1	33.1	32.8	32.7	35.6
65–75 years	22,180	38.8	34.4	32.8	28.9	23.9	34.1	32.5	32.8	31.4	27.4
Education, %											
≤12 years	28,028	39.5	36.0	37.1	39.2	47.7	37.4	35.1	36.7	39.8	50.4
Vocational school/some college	20,247	29.1	28.9	28.9	28.9	28.5	30.2	29.1	29.3	28.7	27.2
≥College graduate	21,098	30.1	34.0	33.1	30.9	22.6	31.3	34.7	33.1	30.6	21.1
Smoking status, %											
Never	21,646	32.4	32.3	30.7	30.9	28.2	32.0	31.7	31.2	30.9	28.6
Former	34,623	49.5	50.2	50.9	49.4	46.9	49.6	50.4	50.7	49.7	46.6
Current	13,049	16.6	16.4	17.3	18.7	23.7	17.2	16.8	17.1	18.4	23.4
Body mass index, %											
<25 kg/m^2^	25,001	36.0	38.9	38.2	35.7	29.7	34.2	38.3	38.0	36.5	31.3
25–<30 kg/m^2^	32,868	47.5	46.7	46.5	46.6	47.0	47.9	46.5	46.3	46.4	47.2
≥30 kg/m^2^	11,904	15.6	14.0	14.9	17.3	22.7	17.1	14.7	15.3	16.7	20.9
Physical activity ^1^, hours	69,227	1.0 ± 1.3	1.2 ± 1.4	1.4 ± 1.5	1.5 ± 1.6	1.6 ± 1.8	1.1 ± 1.3	1.3 ± 1.4	1.4 ± 1.5	1.5 ± 1.6	1.6 ± 1.8
**Women (*n* = 86,292)**	***n***	**(*n* = 17,020)**	**(*n* = 17,309)**	**(*n* = 17,445)**	**(*n* = 17,371)**	**(*n* = 17,147)**	**(*n* = 17,172)**	**(*n* = 17,336)**	**(*n* = 17,369)**	**(*n* = 17,353)**	**(*n* = 17,062)**
Median total energy intake, kcal	86,292	1000.3	1412.7	1769.1	2233.4	3201.0	842.4	1173.6	1451.6	1797.2	2511.1
Race/ethnicity, %											
African American	15,963	25.0	17.3	15.9	15.8	18.6	26.4	17.3	16.0	15.5	17.3
Native Hawaiian	6344	5.0	5.3	5.9	8.1	12.5	5.4	5.5	6.5	7.7	11.7
Japanese American	24,706	26.9	33.2	33.4	29.9	19.7	25.2	33.3	33.0	31.3	20.1
Latino	18,676	20.0	17.3	18.1	20.9	31.9	18.7	16.4	17.6	20.9	34.8
Non-Hispanic white	20,603	23.1	26.9	26.6	25.3	17.3	24.3	27.4	26.9	24.5	16.1
Age groups, %											
45–54 years	30,107	30.4	33.6	34.7	36.1	39.6	34.9	35.0	34.4	34.9	35.4
55–64 years	28,977	33.6	33.2	32.6	33.8	34.8	32.9	33.2	33.1	33.0	35.7
65–75 years	27,208	36.0	33.2	32.7	30.1	25.6	32.2	31.8	32.5	32.2	28.9
Education, %											
≤12 years	38,773	45.5	42.0	41.7	43.9	51.7	43.6	40.9	41.6	44.4	54.3
Vocational school/some college	25,257	29.7	30.1	30.0	29.0	27.5	30.9	30.4	30.3	28.6	26.1
≥College graduate	21,190	23.5	26.7	27.2	25.9	19.4	24.2	27.5	27.0	25.8	18.2
Smoking status, %											
Never	48,255	55.6	56.9	56.4	55.7	55.0	53.2	55.3	57.0	56.8	57.3
Former	24,295	28.2	28.4	28.4	28.6	27.0	29.9	29.5	28.1	28.1	25.3
Current	12,089	14.3	13.0	13.5	13.9	15.3	15.2	13.5	13.4	13.4	14.5
Body mass index, %											
<25 kg/m^2^	40,026	47.8	51.4	50.8	46.0	35.7	45.3	50.5	50.3	47.9	37.8
25–<30 kg/m^2^	27,037	31.2	30.2	30.1	31.8	33.4	31.9	30.2	29.9	31.0	33.6
≥30 kg/m^2^	18,134	19.3	17.3	17.9	21.1	29.4	21.2	18.2	18.9	19.9	27.0
Physical activity ^1^, hours	84,339	0.9 ± 1.1	1.1 ± 1.2	1.1 ± 1.2	1.2 ± 1.3	1.2 ± 1.4	0.9 ± 1.1	1.1 ± 1.2	1.1 ± 1.2	1.2 ± 1.3	1.2 ± 1.4
Menopausal hormone therapy use	83,723	44.2	46.8	46.4	45.9	40.1	43.4	46.6	46.8	46.0	40.6

^1^ Moderate-to-vigorous physical activity.

**Table 2:** Hazard ratios (HRs) (95% confidence intervals (CIs)) for all-causes, cardiovascular disease (CVD), and cancer mortality according to quintiles of total energy intake estimated from a food frequency questionnaire either using an original-portion size (Original-PS) or a gender specific-portion size (GS-PS) among men (*n* = 70,142) in the Multiethnic Cohort Study, 1993–2014 ^1^.

Total Energy Intake	Any Deaths	All-Cause Mortality	CVD Deaths	CVD Mortality	Cancer Deaths	Cancer Mortality
	*n*	HR (95% CI)	*n*	HR (95% CI)	*n*	HR (95% CI)
Original-PS (median)						
Quintile 1 (1243.6 kcal)	5505	1.00 (ref)	1974	1.00 (ref)	1556	1.00 (ref)
Quintile 2 (1749.6 kcal)	5145	1.00 (0.96–1.05)	1776	1.03 (0.96–1.12)	1538	0.99 (0.91–1.08)
Quintile 3 (2191.6 kcal)	4916	0.96 (0.92–1.01)	1686	0.98 (0.91–1.06)	1482	0.94 (0.87–1.02)
Quintile 4 (2748.3 kcal)	4876	1.00 (0.96–1.05)	1674	1.05 (0.97–1.13)	1480	0.97 (0.89–1.05)
Quintile 5 (3877.3 kcal)	5153	1.05 (1.00–1.10)	1744	1.07 (0.99–1.16)	1613	1.03 (0.95–1.13)
*p* for trend		0.0193		0.0844		0.3769
GS-PS (median)						
Quintile 1 (1066.2 kcal)	5278	1.00 (ref)	1888	1.00 (ref)	1538	1.00 (ref)
Quintile 2 (1470.5 kcal)	4974	0.99 (0.95–1.04)	1709	1.02 (0.94–1.10)	1499	0.98 (0.90–1.07)
Quintile 3 (1812.3 kcal)	4907	0.97 (0.92–1.01)	1689	0.99 (0.92–1.07)	1451	0.92 (0.85–1.01)
Quintile 4 (2236.2 kcal)	5057	1.02 (0.97–1.07)	1724	1.05 (0.97–1.13)	1556	1.00 (0.92–1.09)
Quintile 5 (3098.1 kcal)	5379	1.07 (1.02–1.12)	1844	1.11 (1.03–1.20)	1625	1.02 (0.94–1.12)
*p* for trend		0.0010		0.0073		0.3775

^1^ Adjusted for age at cohort entry, race/ethnicity, body mass index, education, physical activity, alcohol intake, and smoking status.

**Table 3:** Hazard ratios (HRs) (95% confidence intervals (CIs)) for all-causes, cardiovascular disease (CVD), and cancer mortality according to quintiles of total energy intake estimated from a food frequency questionnaire either using an original-portion size (Original-PS) or a gender specific-portion size (GS-PS) among women (*n* = 86,292) in the Multiethnic Cohort Study, 1993–2014 ^1^.

Total Energy Intake	Any Deaths	All-Cause Mortality	CVD Deaths	CVD Mortality	Cancer Deaths	Cancer Mortality
	*n*	HR (95% CI)	*n*	HR (95% CI)	*n*	HR (95% CI)
Original-PS (median)						
Quintile 1 (1000.3 kcal)	5348	1.00 (ref)	1917	1.00 (ref)	1402	1.00 (ref)
Quintile 2 (1412.7 kcal)	4729	1.00 (0.95–1.05)	1533	0.94 (0.86–1.02)	1372	1.07 (0.98–1.18)
Quintile 3 (1769.1 kcal)	4747	1.01 (0.96–1.06)	1646	1.01 (0.93–1.10)	1308	1.04 (0.94–1.14)
Quintile 4 (2233.4 kcal)	4610	0.99 (0.94–1.04)	1542	0.96 (0.88–1.05)	1365	1.10 (1.00–1.21)
Quintile 5 (3201.0 kcal)	4699	1.03 (0.98–1.09)	1581	0.99 (0.91–1.08)	1343	1.10 (1.00–1.21)
*p* for trend		0.2704		0.9634		0.0645
GS-PS (median)						
Quintile 1 (842.4 kcal)	5091	1.00 (ref)	1803	1.00 (ref)	1394	1.00 (ref)
Quintile 2 (1173.6 kcal)	4623	1.01 (0.96–1.07)	1504	0.96 (0.88–1.04)	1347	1.06 (0.96–1.16)
Quintile 3 (1451.6 kcal)	4792	1.03 (0.98–1.09)	1653	1.02 (0.94–1.12)	1345	1.06 (0.97–1.17)
Quintile 4 (1797.2 kcal)	4738	1.01 (0.96–1.06)	1579	0.98 (0.90–1.07)	1374	1.07 (0.98–1.18)
Quintile 5 (2511.1 kcal)	4889	1.06 (1.01–1.12)	1680	1.02 (0.94–1.12)	1330	1.07 (0.97–1.18)
*p* for trend		0.0546		0.4698		0.2200

^1^ Adjusted for age at cohort entry, race/ethnicity, body mass index, education, physical activity, alcohol intake, smoking status, and menopausal hormone therapy use.

**Table 4:** Hazard ratios (HRs) (95% confidence intervals (CIs)) for all-causes, cardiovascular disease (CVD), and cancer mortality according to quintiles of percentage energy from macronutrients estimated from a food frequency questionnaire either using an original-portion size (Original-PS) or a gender specific-portion size (GS-PS) among men (*n* = 70,142) in the Multiethnic Cohort Study, 1993–2014 ^1^.

	Any Deaths	All-Cause Mortality	CVD Deaths	CVD Mortality	Cancer Deaths	Cancer Mortality
	*n*	HR (95% CI)	*n*	HR (95% CI)	*n*	HR (95% CI)
Percentage energy from total fat						
Original-PS (median)						
Quintile 1 (22.3%)	4919	1.00 (ref)	1666	1.00 (ref)	1391	1.00 (ref)
Quintile 2 (28.2%)	4873	1.01 (0.96–1.06)	1711	1.06 (0.98–1.15)	1398	0.98 (0.90–1.07)
Quintile 3 (32.0%)	4911	1.07 (1.02–1.12)	1669	1.07 (0.98–1.16)	1511	1.10 (1.01–1.20)
Quintile 4 (35.7%)	5233	1.14 (1.08–1.19)	1794	1.14 (1.05–1.24)	1624	1.15 (1.05–1.25)
Quintile 5 (40.5%)	5659	1.22 (1.16–1.28)	2014	1.27 (1.17–1.38)	1745	1.16 (1.06–1.26)
*p* for trend		<0.0001		<0.0001		<0.0001
GS-PS (median)						
Quintile 1 (23.4%)	4879	1.00 (ref)	1639	1.00 (ref)	1373	1.00 (ref)
Quintile 2 (29.0%)	4868	1.01 (0.97–1.06)	1700	1.07 (0.99–1.16)	1423	1.00 (0.92–1.09)
Quintile 3 (32.6%)	4942	1.06 (1.02–1.12)	1711	1.12 (1.03–1.21)	1493	1.05 (0.96–1.14)
Quintile 4 (36.1%)	5144	1.12 (1.07–1.18)	1781	1.16 (1.07–1.26)	1594	1.10 (1.01–1.20)
Quintile 5 (40.9%)	5762	1.24 (1.18–1.30)	2023	1.30 (1.19–1.41)	1786	1.17 (1.07–1.27)
*p* for trend		<0.0001		<0.0001		0.0001
Percentage energy from carbohydrate						
Original-PS (median)						
Quintile 1 (43.0%)	5609	1.00 (ref)	2009	1.00 (ref)	1679	1.00 (ref)
Quintile 2 (48.6%)	5103	0.94 (0.90–0.98)	1766	0.90 (0.83–0.98)	1605	1.03 (0.95–1.12)
Quintile 3 (52.8%)	4954	0.86 (0.82–0.91)	1657	0.79 (0.73–0.85)	1558	0.99 (0.92–1.08)
Quintile 4 (57.4%)	4941	0.84 (0.80–0.88)	1750	0.83 (0.77–0.90)	1377	0.86 (0.78–0.93)
Quintile 5 (64.3%)	4988	0.81 (0.77–0.85)	1672	0.75 (0.69–0.82)	1450	0.92 (0.84–1.00)
*p* for trend		<0.0001		<0.0001		0.0014
GS-PS (median)						
Quintile 1 (42.7%)	5664	1.00 (ref)	2039	1.00 (ref)	1710	1.00 (ref)
Quintile 2 (48.2%)	5151	0.94 (0.90–0.98)	1758	0.88 (0.81–0.95)	1635	1.03 (0.95–1.11)
Quintile 3 (52.3%)	4935	0.85 (0.81–0.89)	1691	0.80 (0.74–0.87)	1491	0.94 (0.87–1.03)
Quintile 4 (56.5%)	4910	0.83 (0.79–0.87)	1737	0.81 (0.75–0.88)	1424	0.88 (0.81–0.96)
Quintile 5 (62.9%)	4935	0.80 (0.76–0.84)	1629	0.72 (0.66–0.78)	1409	0.90 (0.82–0.98)
*p* for trend		<0.0001		<0.0001		0.0004

^1^ Adjusted for age at cohort entry, race/ethnicity, body mass index, education, physical activity, alcohol intake, smoking status, and total energy intake.

**Table 5:** Hazard ratios (HRs) (95% confidence intervals (CIs)) for all-causes, cardiovascular disease (CVD), and cancer mortality according to quintiles of percentage energy from macronutrients estimated from a food frequency questionnaire either using an original portion size (Original-PS) or a gender-specific portion size (GS-PS) among women (*n* = 86,292) in the Multiethnic Cohort Study, 1993–2014 ^1^.

Women	Any Deaths	All-Cause Mortality	CVD Deaths	CVD Mortality	Cancer Deaths	Cancer Mortality
	*n*	HR (95% CI)	*n*	HR (95% CI)	*n*	HR (95% CI)
Percentage energy from total fat						
Original-PS (median)						
Quintile 1 (21.1%)	4816	1.00 (ref)	1638	1.00 (ref)	1268	1.00 (ref)
Quintile 2 (26.7%)	4750	1.03 (0.98–1.08)	1613	1.04 (0.95–1.13)	1253	0.95 (0.87–1.05)
Quintile 3 (30.6%)	4772	1.05 (1.00–1.10)	1661	1.06 (0.97–1.16)	1359	1.02 (0.93–1.13)
Quintile 4 (34.4%)	4777	1.11 (1.05–1.16)	1586	1.11 (1.02–1.22)	1413	1.09 (0.99–1.20)
Quintile 5 (39.5%)	5018	1.11 (1.05–1.18)	1721	1.17 (1.06–1.28)	1497	1.06 (0.95–1.17)
*p* for trend		<0.0001		0.0006		0.0515
GS-PS (median)						
Quintile 1 (21.6%)	4773	1.00 (ref)	1665	1.00 (ref)	1253	1.00 (ref)
Quintile 2 (26.9%)	4832	1.05 (1.00–1.11)	1612	1.01 (0.92–1.09)	1289	1.02 (0.93–1.13)
Quintile 3 (30.6%)	4718	1.07 (1.02–1.12)	1609	1.07 (0.98–1.16)	1332	1.04 (0.94–1.15)
Quintile 4 (34.2%)	4739	1.09 (1.04–1.15)	1591	1.05 (0.96–1.15)	1406	1.09 (0.99–1.20)
Quintile 5 (39.3%)	5071	1.16 (1.10–1.22)	1742	1.18 (1.08–1.30)	1510	1.13 (1.02–1.24)
*p* for trend		<0.0001		0.0005		0.0088
Percentage energy from carbohydrate						
Original-PS (median)						
Quintile 1 (44.0%)	5090	1.00 (ref)	1765	1.00 (ref)	1499	1.00 (ref)
Quintile 2 (49.9%)	4727	0.91 (0.86–0.96)	1612	0.88 (0.80–0.96)	1380	0.95 (0.86–1.05)
Quintile 3 (54.2%)	4774	0.90 (0.86–0.95)	1610	0.87 (0.79–0.95)	1396	0.95 (0.86–1.05)
Quintile 4 (58.7%)	4697	0.85 (0.81–0.90)	1567	0.81 (0.74–0.89)	1245	0.83 (0.75–0.92)
Quintile 5 (65.3%)	4845	0.85 (0.81–0.90)	1665	0.81 (0.74–0.89)	1270	0.90 (0.81–1.00)
*p* for trend		<0.0001		<0.0001		0.0056
GS-PS (median)						
Quintile 1 (44.5%)	5070	1.00 (ref)	1778	1.00 (ref)	1480	1.00 (ref)
Quintile 2 (50.4%)	4734	0.92 (0.87–0.97)	1591	0.89 (0.81–0.97)	1420	0.99 (0.89–1.09)
Quintile 3 (54.5%)	4729	0.89 (0.84–0.94)	1567	0.83 (0.76–0.91)	1344	0.93 (0.84–1.03)
Quintile 4 (58.7%)	4719	0.86 (0.82–0.91)	1608	0.83 (0.75–0.90)	1256	0.87 (0.79–0.97)
Quintile 5 (64.9%)	4881	0.85 (0.81–0.90)	1675	0.81 (0.74–0.88)	1290	0.91 (0.82–1.00)
*p* for trend		<0.0001		<0.0001		0.0095

^1^ Adjusted for age at cohort entry, race/ethnicity, body mass index, education, physical activity, alcohol intake, smoking status, menopausal hormone therapy use, and total energy intake.
